# Effects of external Chinese herbal therapy combined with pulse electromagnetic field on older adults with knee osteoarthritis: study protocol for a randomized controlled trial

**DOI:** 10.3389/fmed.2025.1498622

**Published:** 2025-05-22

**Authors:** Xiang Li, Foxiao Li, An Yu, Qi Bu, Ruirui Wang, Lijing Li, Wei Zhang, Lei Yang

**Affiliations:** ^1^Department of Rehabilitation Medicine, The Second People's Hospital of Kunming, Kunming, Yunnan, China; ^2^Department of Rehabilitation Medicine, Yan’an Hospital of Kunming City, Kunming, Yunnan, China; ^3^Yunnan Key Laboratory for Basic Research on Bone and Joint Diseases, Kunming University, Kunming, Yunnan, China

**Keywords:** osteoarthritis, herbs, pulsed electromagnetic field, pain, function, study protocol, randomized controlled trial

## Abstract

**Introduction:**

Knee osteoarthritis (KOA) is a major cause of disability, and both external Chinese herbal therapy (ECHT) and pulsed electromagnetic field (PEMF) therapy have shown potential in managing KOA symptoms. However, no research has directly compared the efficacy of ECHT, PEMF, and their combined use on pain relief and functional improvement in older adults with KOA. This highlights a significant research opportunity to explore the comparative benefits of these treatment modalities.

**Methods and analysis:**

This randomized controlled trial (RCT) aims to enroll 124 older adults with KOA and will randomly allocate them across four treatment groups: (1) exercise with health education, (2) exercise with ECHT, (3) exercise with PEMF therapy, and (4) exercise with a combination of ECHT and PEMF. Each intervention will be administered five times per week for 4 weeks. The primary endpoints are pain and functional capacity, while secondary measures encompass lower limb range of motion, alignment, joint space, and serum cytokine levels. Assessments will be conducted at baseline, post-intervention, and at the 4-week follow-up.

**Discussion:**

This trial is poised to conduct a thorough comparative analysis of the impacts of ECHT, PEMF therapy, and their combined application in older adults with KOA. The study findings will not only inform clinical practice by offering data-driven recommendations but also enrich the scientific discourse on the complex interplay between inflammation, pain, and function in KOA.

**Clinical trial registration:**

Chinese clinical trial registry platform: www.chictr.org.cn (no. ChiCTR2400087644).

## Introduction

Knee osteoarthritis (KOA) stands as the world’s most prevalent musculoskeletal disorder and a leading cause of disability, affecting millions globally (1). Common symptoms of KOA include pain, stiffness, and impaired functionality, significantly impacting the quality of life (2).

KOA affects the entire joint architecture, which leads to structural changes across multiple components: hyaline articular cartilage, subchondral bone, ligaments, joint capsule, synovium, and periarticular muscles, reflecting its complexity (3, 4). The intricate pathogenesis of KOA is influenced by a confluence of factors—mechanical, inflammatory, and metabolic—that culminate in the deterioration and eventual failure of the synovial joint, perpetuating a detrimental cycle.

The personal, social, and economic toll of KOA is extensively documented, underscoring the pressing need for precise and efficacious therapeutic interventions to mitigate these burdens (5, 6). Despite significant strides in our comprehension of KOA, the development of impactful treatments remains a formidable challenge (7, 8). The current armamentarium for KOA management spans a diverse array of strategies, including non-pharmacological interventions, pharmacological therapies, and surgical interventions (9). The overarching objectives of these treatments are to alleviate symptoms and to slow the progression of the disease.

Clinical guidelines prioritize first-line treatments that encompass patient education, self-management strategies, regular exercise, weight management for overweight individuals, and physical therapy (10, 11). Although pharmacotherapy can provide symptomatic relief for KOA, the potential for adverse effects can limit the widespread use of these medications, highlighting the imperative for continuous research into safer and more tolerable treatment options (12, 13). It is essential to explore innovative approaches that can enhance the therapeutic landscape for KOA, ensuring that patients have access to treatments that are both effective and safe.

The escalating apprehensions regarding the safety profiles and economic implications of conventional KOA treatments have spurred a burgeoning interest in the exploration of natural remedies as credible alternatives. This shift is further compounded by the enduring challenge of managing the chronic pain associated with KOA, which has led to an intensified investigation into the potential of herbal therapies as viable solutions (8).

The exploration of herbal remedies in the treatment of KOA has unveiled innovative therapeutic pathways, offering fresh perspectives on managing this complex condition (14, 15). Among these, the topical external Chinese herbal therapy (ECHT) has risen to prominence, capturing the interest of both patients and healthcare providers due to its unique approach to KOA management (16).

ECHT’s initial indications of efficacy are promising, yet they underscore the necessity for a more profound and methodical investigation. To solidify its place within the KOA therapeutic framework, ECHT requires extensive research and well-designed clinical trials. These studies should not only validate its safety and effectiveness but also delve into the intricate biological mechanisms that confer its therapeutic benefits.

By understanding how ECHT interacts with the body’s systems and influences the disease process, researchers can refine its application, potentially enhancing its efficacy and safety profile. This meticulous scientific exploration is vital for establishing ECHT as a reliable and evidence-based component of KOA treatment strategies. It will also empower healthcare providers to make informed decisions when recommending ECHT to patients, ensuring that they can do so with a clear understanding of its potential benefits and any associated risks.

On the other hand, pulsed electromagnetic field (PEMF) therapy has gained attention as a simple, safe, and noninvasive treatment option for KOA (17). Its appeal lies in its potential to stimulate bone and cartilage repair, offering relief from pain and stiffness (18, 19). Recent guidelines have acknowledged PEMF’s therapeutic value; however, the endorsement is cautiously moderate due to the relatively short-term follow-ups in the existing body of research. There is an evident requirement for long-term studies to confirm and expand upon PEMF’s therapeutic potential (20).

Furthermore, the pathogenesis of KOA involves a complex interplay of factors. Current research indicates that joint stress injuries and apoptosis contribute to the degeneration of articular cartilage, while an imbalance in chondrocyte synthetic metabolism can lead to abnormal immune responses, which are central to the disease’s progression (21). Experimental studies have identified key players in this process, such as interleukin (IL)-1 and matrix metalloproteinase (MMP), which significantly influence the development and worsening of KOA (22).

When the knee joint cartilage sustains damage, the release of pro-inflammatory cytokines, including tumor necrosis factor (TNF-*α*), IL-1, IL-6, and nitric oxide (NO), into the synovial fluid initiates local inflammatory responses (21). This inflammatory activation triggers multiple signaling pathways, leading to the production of matrix metalloproteinase-3 (MMP-3) and inducible nitric oxide synthase (iNOS). The ensuing imbalance between matrix metalloproteins (MMPs) and tissue inhibitors of metalloproteinases (TIMPs) disrupts the delicate equilibrium within the articular cartilage (23). This disruption compromises chondrocyte function and the structural integrity of the extracellular matrix, hastening the degradation of the cartilage matrix and thus contributing to the initiation and progression of OA. Understanding these intricate mechanisms is crucial for developing targeted therapies that can address the root causes of KOA, potentially halting or even reversing the disease’s progression. As research continues to unravel the complexities of KOA pathogenesis, the hope is to identify more effective interventions that can improve patient outcomes and quality of life.

Our comprehensive review of the literature reveals a significant gap in studies examining the synergistic effects of ECHT and PEMF therapy on pain reduction and functional improvement in elderly KOA patients. The objective of this study is to address this research gap by investigating the impacts of ECHT therapy, PEMF therapy, and their combined application on pain alleviation, functional enhancement, and molecular changes in older adults with KOA. This study aims to investigate the individual and combined effects of ECHT and PEMF therapies on pain alleviation and functional improvement in older adults with KOA and gather evidence that elucidates the underlying molecular mechanisms at play, providing a deeper understanding of how these therapies influence the disease’s progression at a molecular level.

We hypothesize that ECHT and PEMF therapies will each induce distinct improvements in pain and functionality, as well as varying molecular responses in older adults with KOA. We anticipate that the combined application of both therapies will result in more pronounced molecular changes, potentially leading to superior therapeutic outcomes compared to either treatment alone.

This research is poised to contribute valuable insights into the comparative efficacy of these therapies and their potential synergistic effects, thereby informing more personalized and effective treatment strategies for older adults suffering from KOA.

## Methods and analysis

### Study design

**Figure 1:**
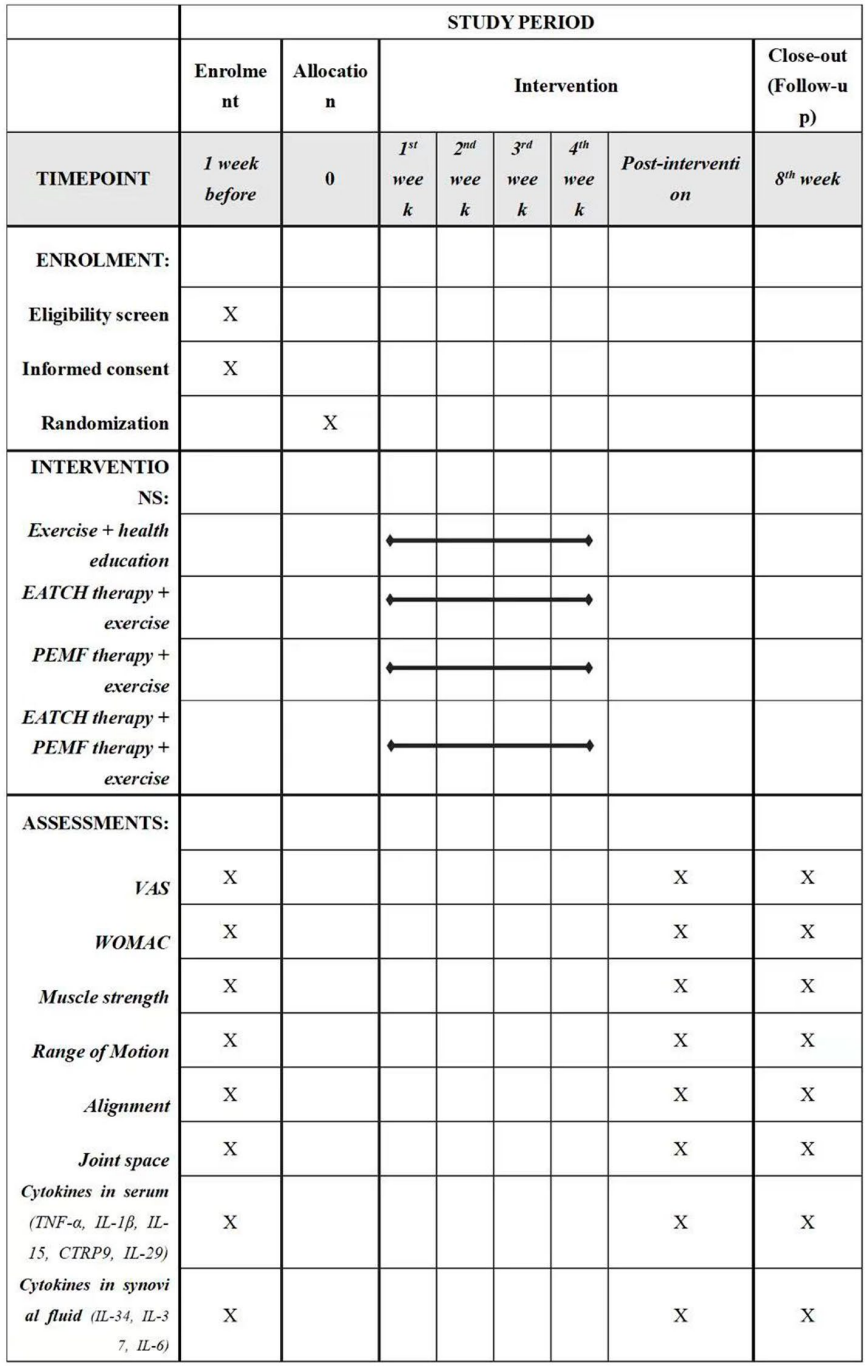
Schedule of enrolment, interventions, and assessments. EATCH, The external application of traditional Chinese herbs; PEMF, pulsed electromagnetic field; VAS, visual analogue scale; WOMAC, The Western Ontario and McMaster Universities Osteoarthritis Index (Likert version 3.11).

**Figure 2:**
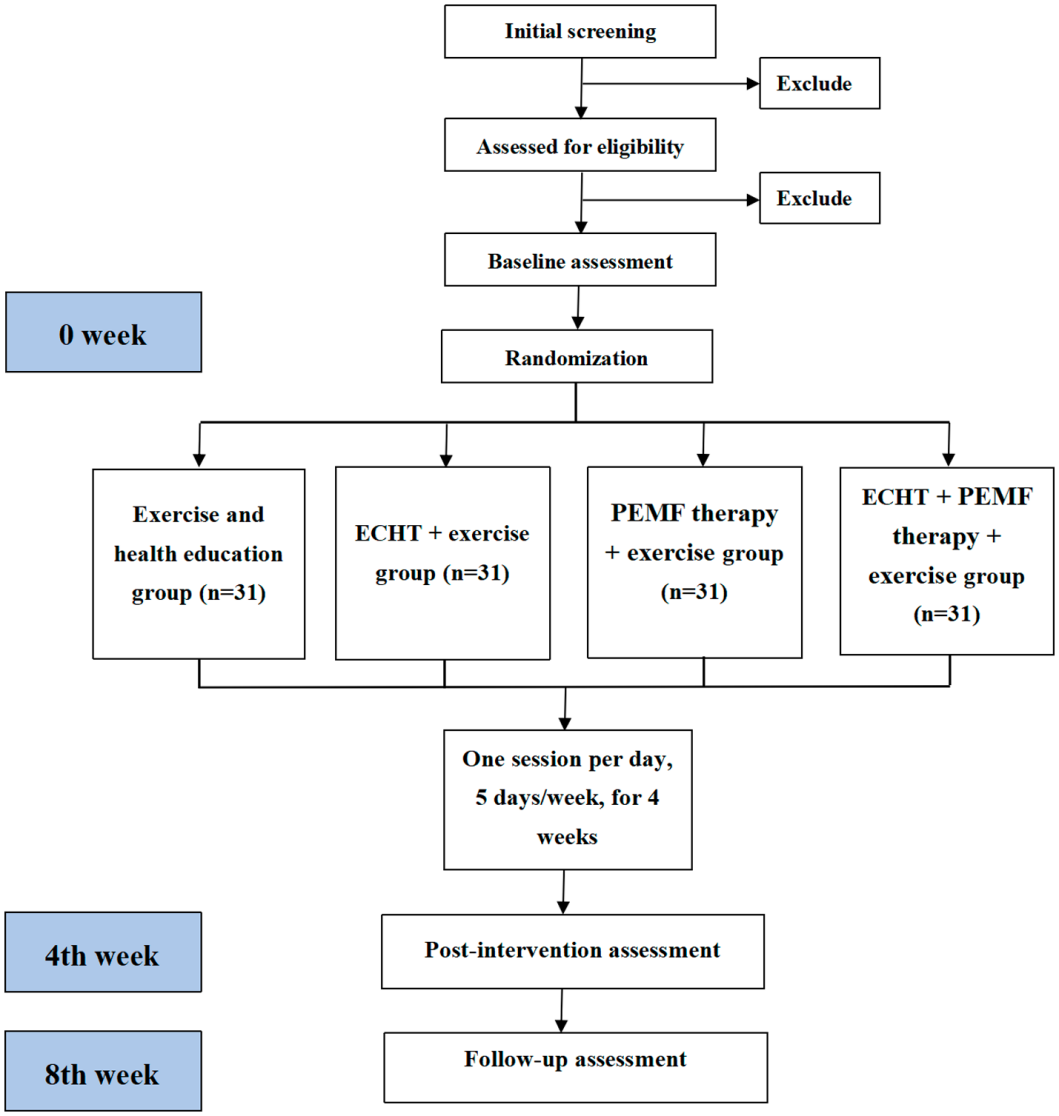
Study flowchart of the research process according to the randomized trial synthesis criteria. ECHT, external Chinese herbal therapy; PEMF, pulsed electromagnetic field.

This will be a prospective, single-blinded, parallel-armed, randomized controlled trial (RCT). Participants will be allocated to one of the four groups: (1) exercise and health education group, (2) ECHT therapy + exercise group, (3) PEMF therapy + exercise group, or (4) ECHT + PEMF therapy + exercise group. The checklist of Standard Protocol Items: Recommendations for Interventional Trials (SPIRIT) is provided in Appendix 1, while the SPIRIT schedule is presented in Figure 1. Figure 2 illustrates the flowchart detailing the progression of the trial’s methodology.

To explore the molecular mechanisms of ECHT, PEMF, and their combined therapy in older adults with KOA, this study will employ molecular biological techniques to analyze the following aspects:

Assessment of inflammatory markers: enzyme-linked immunosorbent Assay (ELISA) will be employed to meticulously measure variations in the serum levels of key inflammatory response factors. Specifically, it detects alterations in C-reactive protein (CRP), high-sensitivity C-reactive protein (hs-CRP), TNF-*α*, and interleukin-6 (IL-6), providing a quantitative assessment of the inflammatory state in patients with KOA (24).

Exploration of inflammatory pathways: a multifaceted approach will be utilized to investigate the intricate mechanisms of inflammation in KOA.

Immunoturbidimetry: this technique will be applied to quantify the serum levels of Matrix Metalloproteinase-3 (MMP-3), Matrix Metalloproteinase-13 (MMP-13), and hyaluronic acid (HA), which are pivotal in the degradation of cartilage (25).

Colorimetric methods: these will be employed to assess the serum content of NO, and malondialdehyde (MDA), offering insights into oxidative stress and the body’s antioxidant defenses.

ELISA: Will be further utilized to gauge the levels of Interleukin-1 beta (IL-1β), prostaglandin E2 (PGE-2), and IL-1, which are critical mediators of the inflammatory response in KOA (26).

### Participants

Participants will be recruited from The Second People’s Hospital of Kunming, and Yan’an Hospital of Kunming City. The trial was registered in the www.chictr.org.cn (ChiCTR2400087644), and will be conducted in accordance with the Declaration of Helsinki. All participants will provide written informed consent before data collection. The trial has begun on July 22, 2024, and will be end on May 30, 2025.

The inclusion criteria are as follows: (1) aged between 60 and 80 years old; (2) a diagnosis of KOA in at least one knee, confirmed by the American College of Rheumatology’s clinical criteria; (3) a Kellgren-Lawrence (K-L) radiographic grade of 2 or higher. Exclusion criteria included: (1) the presence of a neurodegenerative or neurosensory disorder affecting the knee, distinct from KOA; (2) a history of lower extremity joint trauma within the last 3 months; (3) a scheduled total knee replacement in the upcoming months; (4) a documented allergy to traditional Chinese herbs; (5) prior treatment for KOA with steroids, intra-articular injections, or other analgesics within the past 3 months; (6) concomitant other physical therapies.

### Randomization and blinding

The random allocation sequence will be generated by an online generator (https://www.random.org). The allocation sequence will be kept in a sealed, opaque envelope. The principal investigator will assign participants to the respective intervention groups. The allocation will be blinded to the outcome assessors and the data analyst. Participants will be instructed not to disclose the allocation to the outcome assessors.

### Interventions

Based on the research intervention description of the TIDieR template (27), details of the intervention protocol for the four groups are provided in Table 1. Interventions will be taken place in the physical therapy room. Participants in all four groups will receive a daily intervention, structured as five sessions per week for 4 weeks. Each session will start with a 30-min routine exercise, followed by the therapeutic intervention tailored to their group assignment: either ECHT therapy, PEMF therapy, a combination of both, or health education.

**Table 1:** The training protocol for each group base on the TIDieR template.

Item	Exercise and health education group	External application of traditional Chinese herbs therapy + exercise group	Pulsed electromagnetic field therapy + exercise group	External application of traditional Chinese herbs + Pulsed electromagnetic field + exercise group
1. Brief name	Ex + HE	EATCH + Ex	PEMF + Ex	EATCH + PEMF therapy + Ex
2. Why	1. Exercise therapy relieves knee pain at all stages of OA. Bennell et al.’s (28) study showed that a greater benefit for pain was found with a non-weight bearing quadriceps exercise program than with a weight bearing program in people with obesity. Therefore, a non-weight bearing quadriceps exercise program was adopted in our trial. 2. Patient education is vital to improve the patients’ understanding of their current condition and to ensure compliance to exercise program and long-term self-management at home.	1. The reason why to perform exercise is the same as shown in group 1. 2. Herbs have demonstrated strong anti-inflammatory and antioxidant properties, which can mitigate inflammation and reduce tissue damage. However, further research and clinical trials are essential to establish its safety, efficacy, and to explore its underlying mechanisms.	1. The reason why to perform exercise is the same as shown in group 1. 2. Recent guidelines recognize PEMF as an effective therapy for bone and cartilage pathology, capable of alleviating pain and stiffness. However, current recommendation for PEMF is moderate, primarily due to the limited duration of follow-ups in existing studies. There is a clear need for long-term studies to solidify its therapeutic potential.	1. The reason why to perform exercise is the same as shown in group 1. 2. No study has explored the combined impact of EATCH and PEMF therapy on pain alleviation and functional improvement in older adults with KOA.
3. What materials	Sand bags (ankle cuff, 0.5–5 kg) and elastic resistance bands with various color.	1. Sand bags (ankle cuff, 0.5 kg to 5 kg) and elastic resistance bands with various color. 2. Compound herbs powder, sterile gauze pad, and elastic bandages.	1. Sand bags (ankle cuff, 0.5 kg to 5 kg) and elastic resistance bands with various color. 2. The Pulsed Electromagnetic Field Therapy Device by Haobro Company, Suzhou, China.	1. Sand bags (ankle cuff, 0.5 kg to 5 kg) and elastic resistance bands with various color. 2. Compound herbs powder (as in group 2), sterile gauze pad, and elastic bandages. 3. The Pulsed Electromagnetic Field Therapy Device by Haobro Company, Suzhou, China.
4. Procedures	Participants will start the program with two sets of 10 repetitions for each exercise for the first 1 week and progress to three sets thereafter or as quickly as able. Participants will be instructed that each exercise should be performed slowly in a controlled manner. Progression will be guided by the physiotherapist at regular intervals with adjustments to participants’ ankle weights or elastic resistance bands. Participants will also be encouraged to increase the weights (0.5 kg at a time) for each exercise if they feel the exercise is “easier to complete” compared to the beginning of that week. The end position of each exercise is to be held initially for 5 s and then increased to 10 s.	The compound herbs will be finely ground into a uniform powder and thoroughly mixed. For each treatment session, 5 grams of this powder will be combined with a small amount of warm water to form a smooth, pliable paste. Then, this paste will be evenly spread onto a sterile gauze pad. The therapists will identify the most painful area of the patient’s knee, disinfect the site using 75% ethanol-soaked cotton balls, and apply the medicated gauze directly onto the affected point, securing it with elastic bandages. The application of the gauze and bandages will be kept in place for 6–8 h daily, and the local skin area will be gently cleansed with water during dressing changes.	Participants will be positioned either supine or in a long-sitting position on the treatment bed for PEMF therapy. The magnetic field will be induced using two pairs of solenoid applicators from the PEMF device. These applicators will be securely fixed to the sides of the knee with a velcroband. During each session, the PEMF parameters will be set to a frequency of 50 Hz, an intensity of 0.8 Tesla, and each session will last for 30 min. Patients will be informed that the device would not produce any noticeable noise or sensations.	Participants will perform exercises first, next receive EATCH and PEMF therapy for 30 min simultaneously. Then, The application of the gauze and bandages for EATCH will be kept in place for 6–8 h as in group 2.
5. Who provided	Experienced physiotherapists	Experienced physiotherapists	Experienced physiotherapists	Experienced physiotherapists
6. How	Exercise will be performed with a therapist: participant ratio of 1:2.	Participants will perform exercise first. Next, receive the EATCH.	Participants will perform exercise first. Next, receive the PEMF therapy.	Exercise will be performed with a therapist: participant ratio of 1:2.
7. Where	The exercise will be performed in the PT room of The Second People’s Hospital of Kunming, and the PT room of Yan An Hospital of Kunming City.	The exercise and EATCH will be performed in the PT room of The Second People’s Hospital of Kunming, and the PT room of Yan An Hospital of Kunming City.	The exercise and PEMF therapy will be performed in the PT room of The Second People’s Hospital of Kunming, and the PT room of Yan An Hospital of Kunming City.	The exercise and combined therapy (EATCH + PEMF) will be performed in the PT room of The Second People’s Hospital of Kunming, and the PT room of Yan An Hospital of Kunming City.
8. When and how much	This group will perform the strengthening exercise 30 min/day first, then receive health education for 30 min. The intervention will last 5 days/week, for 4 weeks.	This group will perform the strengthening exercise 30 min/day first, then receive the EATCH therapy. The intervention will last 5 days/week, for 4 weeks.	This group will perform the strengthening exercise 30 min/day first, then receive the PEMF therapy for 30 min. The intervention will last 5 days/week, for 4 weeks.	This group will perform the strengthening exercise 30 min/day first, then receive the EATCH and PEMF therapy simultaneously for 30 min. The intervention will last 5 days/week, for 4 weeks.
9. Tailoring	The exercises of this group can be tailored to the capacity of the participants, including: resistance, repetitions, and sets.	The exercises of this group can be tailored to the capacity of the participants, including: resistance, repetitions, and sets. The application of the gauze and bandages will be depend on the affected point of the knee.	The exercises of this group can be tailored to the capacity of the participants, including: resistance, repetitions, and sets.	The exercises of this group can be tailored to the capacity of the participants, including: resistance, repetitions, and sets.
10. How well	The assessors will be blinded to the group allocation, in order to ensure the objectivity and impartiality of the assessments.	The assessors will be blinded to the group allocation, in order to ensure the objectivity and impartiality of the assessments.	The assessors will be blinded to the group allocation, in order to ensure the objectivity and impartiality of the assessments.	The assessors will be blinded to the group allocation, in order to ensure the objectivity and impartiality of the assessments.

#### Exercise and health education group

The exercise and health education group will serve as an active control group, enabling us to determine whether the observed changes in the ECHT therapy, PEMF therapy, or their combination are a function of maturation or repeated testing. This exercise regimen, detailed in Supplementary Figure 1, was adapted from established protocols of prior studies and focused on non-weight-bearing movements in sitting or supine positions to minimize stress on the affected lower limb (28, 29). Next, participants in this group will receive an additional 30 min of health education on KOA.

#### ECHT + exercise group

**Figure 3:**
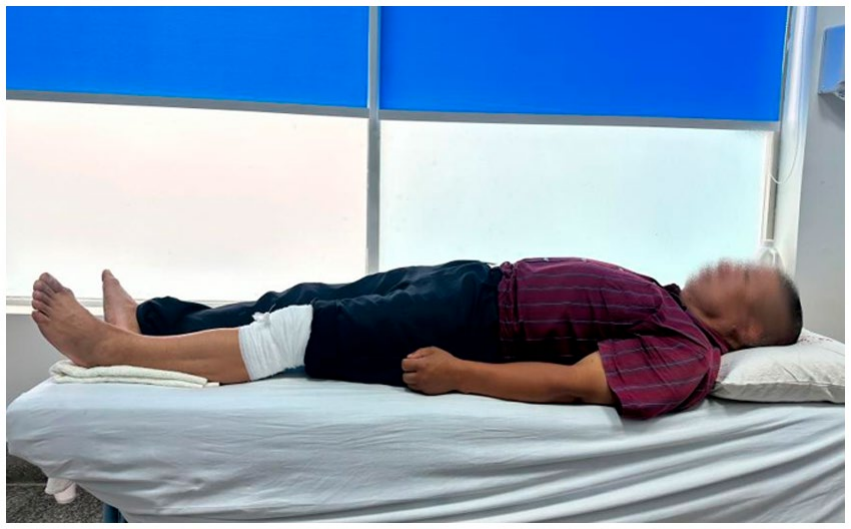
External Chinese herbal therapy (ECHT).

The compound prescription of traditional Chinese herbs was developed in accordance with the principles of the “Inner Canon of the Yellow Emperor” and will be supplied by the Second People’s Hospital of Kunming. Details regarding the specific types and quantities of the herbs used for the external application are outlined in Table 2. These herbs will be finely ground into a uniform powder and thoroughly mixed. For each treatment session, 5 grams of this powder will be combined with a small amount of warm water to form a smooth, pliable paste. Then, this paste will be evenly spread onto a sterile gauze pad. The therapists will identify the most painful area of the patient’s knee, disinfect the site using 75% ethanol-soaked cotton balls, and apply the medicated gauze directly onto the affected point, securing it with elastic bandages. The application of the gauze and bandages will be kept in place for 6–8 h daily, and the local skin area will be gently cleansed with water during dressing changes (Figure 3).

**Table 2:** Types and quantities of the herbs used for the external application.

Types	Quantity (gram)
Pseudo-ginseng	30
Radix rehmanniae	30
Ginseng	30
Liquorice	60
Angelica sinensis	60
*Polygonum multiflorum*	30
Lotus seed	30
*Sedum sarmentosum*	60
Cattail pollen	60
Lotus rhizome node	15
Sappanwood	30
Dragon’s blood	30
Tianzhu Yellow	50
Fossil fragments	30
Albizia bark	30
Caulis polygoni multiflori	20
Rhizoma alismatis	30
American ginseng	20
Tortoise plastron	60
Glutinous rice roots and whiskers	30

#### PEMF therapy + exercise group

**Figure 4:**
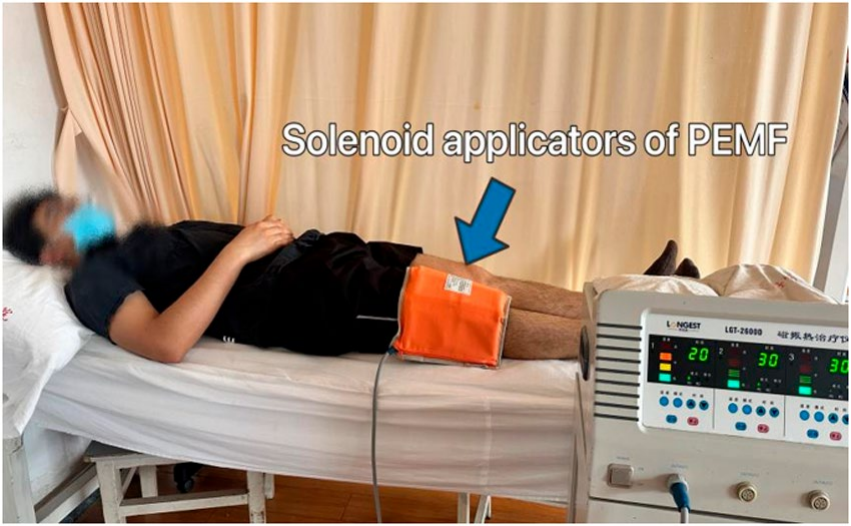
Pulsed electromagnetic field (PEMF) therapy.

Participants will be positioned either supine or in a long-sitting position on the treatment bed for PEMF therapy. The magnetic field will be induced using two pairs of solenoid applicators from the Pulsed Electromagnetic Field Therapy Device (Model: HB240D) by Haobro Company, Suzhou, China (Figure 4). These applicators will be securely fixed to the sides of the knee with a velcroband. During each session, the PEMF parameters will be set to a frequency of 50 Hz, an intensity of 0.8 Tesla, and each session will last for 30 min. Patients will be informed that the device would not produce any noticeable noise or sensations.

#### ECHT + PEMF therapy + exercise group

**Figure 5:**
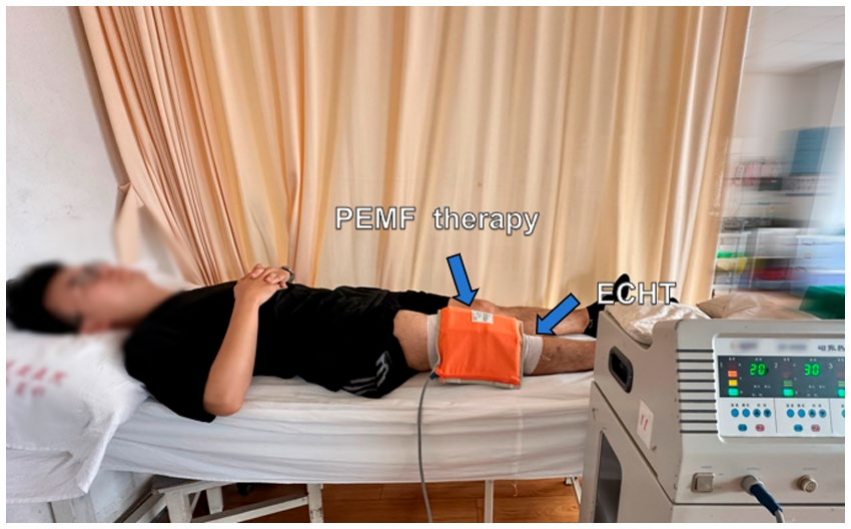
Combined external Chinese herbal therapy (ECHT) and pulsed electromagnetic field (PEMF) therapy.

After the 30-min routine exercise, participants in this group will receive ECHT and PEMF therapy for 30 min simultaneously (Figure 5). Then, the application of the gauze and bandages for ECHT will be kept in place for 6–8 h as in the ECHT group.

### Outcome measures

All demographic information (e.g., age, gender and affected side, etc.) will be collected at baseline assessment session. All outcome assessments will be performed by a blinded assessor in each hospital on three occasions, i.e., at baseline, within 1-week after the completion of intervention, and at a 4-week follow-up.

#### Primary outcomes

*Pain:* overall average pain severity in the past week will be measured via an 11-point visual analog scale (VAS) with terminal descriptors of “no pain” (score 0) and “worst pain possible” (score 10) (30). The minimum clinically important difference of NPRS was shown to be 1.8 (31, 32).

*Overall function:* the Western Ontario and McMaster Universities (WOMAC) Osteoarthritis Index (Likert version 3.1) will be used to measure the overall function in the past week (33). A total of 24 questions are divided in three categories assessing pain, stiffness and physical function, with Likert response options ranging from 0 (none), 1 (mild), 2 (moderate), 3 (severe), to 4 (very severe). A higher score indicates more intense pain, stiffness and physical dysfunction. Though WOMAC is a self-report, disease-specific instrument, excellent test–retest reliability (ICC = 0.83) and convergent validity (Pearson’s r = 0.77) have been demonstrated (34, 35).

#### Secondary outcomes

*Muscle strength*: the manual muscle test (MMT) will be used to test the muscle strength of flexor and extensor in knee and hip.

*Range of Motion (ROM)*: the goniometer will be used to measure the active and passive flexion and extension ROM in hip, knee, and ankle joint.

*Alignment:* the Q angle will be measured via the X-ray.

*Joint space:* the joint space will be quantified by X-ray.

*Cytokines in serum:* TNF-*α*, interleukin-1β (IL-1β), interleukin-15 (IL-15), C1q/tumor necrosis factor-related protein-9 (CTRP9), and interleukin-29 (IL-29), CRP, hs-CRP, MMP-3, MMP-13, HA, NO, MDA, PGE-2.

*Cytokines in synovial fluid:* interleukin-34 (IL-34), interleukin-37 (IL-37), and interleukin-6 (IL-6).

### Sample size

GPower 3.1 software (Heinrich-Heine-Universitat, Dusseldorf, Germany) was used to estimate the sample size. Wang et al. (36) 8-week traditional Chinese herbs hot compress (TCHHC) trial demonstrated a significant interaction on the total WOMAC score between the TCHHC combined with therapeutic exercise group and the therapeutic exercise alone group, yielding a partial eta-squared ( $\eta_{p}^{2}$ ) of 0.148, corresponding to an effect size of *f* = 0.416 (36). A conservative approach was adopted for our sample size estimation, considering the comparison of two distinct treatment factors—ECHT and PEMF—and their combined application, alongside a reduced intervention duration of 4 weeks. Employing a 4 (group factor) × 3 (time factor) RM ANOVA framework, and assuming a small effect size (*f* = 0.15), a significance level (*α*) of 0.05, statistical power of 0.80, and accounting for a 15% attrition rate, the total sample size required was 124 participants, evenly distributed across the four groups, i.e., 31 in each group. The sample size is expanded by 10% compared to the original registration in the Chinese Clinical Trial Registry. This modification was necessitated by a post-initiation adjustment to the attrition rate assumption, which was revised upward to a more conservative 15% based on emerging data from the ongoing trial.

### Withdrawal and adverse events

All the intervention methods in this trial are very safe, however, if participants show irreversible intervention-related adverse events during the trial, such as nausea, dizziness, disorientation, or fatigue, the trial will be stopped by the participant and registered. Moreover, this study will strictly comply with the clinical operation and internationally recognized legal requirements. Any adverse events will be reported directly to the ethics committee immediately, and the intervention regimen will be adjusted accordingly after each adverse event. There is no anticipated harm or compensation for trial participation. In addition, the following measures will be undertaken to achieve the best intervention environment: (1) during the 4-week intervention, an individualized intervention plan will be made for each participant on a weekly basis, and the intervention schedule will be adjusted according to the actual conditions of the participant; (2) close attention will be paid and regular feedback will be given to the participants during intervention; (3) the intervention effect on participants during the intervention will be evaluated weekly; and (4) participants’ decision to withdraw from the study will be supported.

### Statistical analysis

Data entry will be performed by two independent researchers so that the accuracy of the input data can be cross-checked. The Statistical Package for Social Sciences (SPSS) version 26 will be used to conduct the data analysis. Intention-to-treat analysis will be performed. The normality of the data will be checked using the Shapiro–Wilk test. To compare the baseline characteristics of the four groups, one-way ANOVA (or its nonparametric equivalent: Kruskal–Wallis test) or Chi-square tests will be used, depending on whether the criteria for parametric statistics are fulfilled. Those relevant variables that show important between-group differences will be entered as covariates for subsequent analysis. To compare the treatment effects of the 4 intervention approaches, multivariate 4 × 3 ANOVA or ANCOVA with repeated measures (mixed design; repeated factor: time; between-subject factor: group) will be used to determine the time effect and treatment × time interaction effect for each outcome variable of interest. The Greenhouse–Geisser correction will be applied if the assumption of sphericity is violated. If significant results are found, *post-hoc* multiple comparisons of group effects and contrast analyses of time effects will be performed. Bonferroni adjustment will be performed in *post-hoc* analysis to account for the increased risk of Type I errors associated with multiple comparisons. If the criteria for parametric statistics are not fulfilled, the Mann–Whitney test and Wilcoxon test will be used.

### Data management and monitoring

The safety supervision committee of the involved hospitals will oversee this project and evaluate the experimental design, scientific rigor, participant safety, adherence to medical ethics, and data management. Strict measures will be implemented to safeguard the privacy rights of each participant. Initial data and outcome indicators will be securely stored in a highly protected database, and anonymization procedures will be rigorously followed to ensure the safety and confidentiality of all participants.

All patients’ data will be archived in a unique confidential manner at all stages of the trial and also this confidentiality will be protected during and after the trial.

### Dissemination

The findings of this trial will be submitted to peer-reviewed journals and academic conferences, and will be reported according to the CONSORT guidelines (37).

## Discussion

This trial is poised to conduct a thorough comparative analysis of the impacts of ECHT, PEMF therapy, and their combined application on pain alleviation, functional improvement, and the modulation of blood inflammatory biomarkers in older adults with KOA. The study is meticulously designed to test the central hypothesis that a synergistic approach, integrating both ECHT and PEMF therapies, will induce more pronounced alterations in blood inflammatory biomarkers. This, in turn, is hypothesized to result in superior improvements in pain and functional capabilities compared to either intervention employed in isolation.

The study’s objectives are twofold: (1) to rigorously evaluate whether the integration of ECHT and PEMF therapy can achieve more significant modulation of inflammatory biomarkers, which are pivotal in the pathophysiology of KOA. (2) To determine if this combined therapeutic strategy leads to enhanced clinical outcomes in terms of pain reduction and functional enhancement in the elderly population suffering from KOA.

The implications of this trial are profound, as it seeks to provide evidence-based guidance for intervention strategies tailored to the unique needs of older adults with KOA. By elucidating the potential synergistic effects of ECHT and PEMF therapy on inflammatory pathways, the study aims to contribute to a deeper understanding of the mechanisms underlying pain relief and functional improvement in KOA. This knowledge is critical for advancing the development of personalized treatment plans that are both effective and safe for this vulnerable patient demographic.

Ultimately, the findings of this study will not only inform clinical practice by offering data-driven recommendations but also enrich the scientific discourse on the complex interplay between inflammation, pain, and function in KOA. The pursuit of these insights is essential for advancing the frontiers of OA management and enhancing the quality of life for older adults affected by this debilitating condition.

## Trial status

The recruitment of this trial has begun on August 5, 2024, and will be completed on April 30, 2025.
