# Supplementary material for: Effects of external Chinese herbal therapy combined with pulse electromagnetic field on older adults with knee osteoarthritis: study protocol for a randomized controlled trial
Source: Front Med (Lausanne). 2025 May 22;12:1498622. doi: 10.3389/fmed.2025.1498622 (PMC12137307; doi:10.3389/fmed.2025.1498622)
Supplement: Supplementary file 1 [file Table_1.docx]

**Appendix 1 - Non-weightbearing quadriceps strengthening exercise program**

**1. Quads over a roll (inner range knee extension)**

Starting position:

Put weight on the arthritic leg's ankle.

Sit on a stable surface, using your arms behind you to support your body weight. If preferred, you may lie down. Bend your non-study leg and use several rolled-up towels to support your arthritic knee. Ensure that your arthritic knee is bent at approximately 30°.

Pull up the foot and ankle on the arthritic leg, keeping the kneecap and toes pointed to the ceiling.

Exercise:

Keeping the knee in touch with the towel, SLOWLY pull the heel off the surface to straighten the arthritic leg.

**
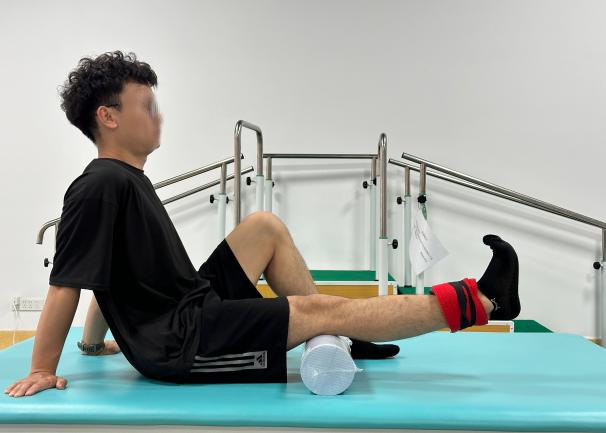
**

**
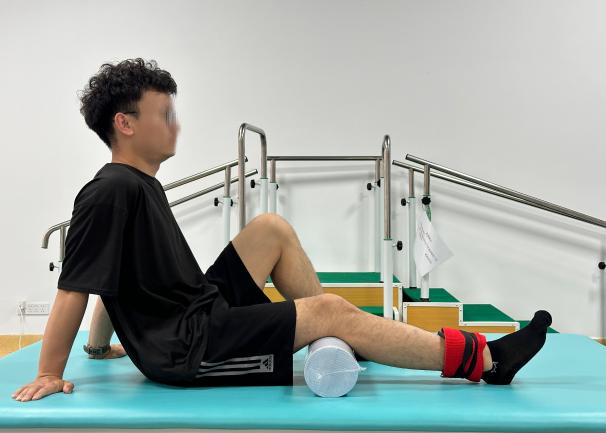
**

**2. Knee extension in sitting**

Starting position:

Put weight on the arthritic leg's ankle.

With both legs raised off the floor, take a seat over the edge of a chair or bed. You can place some books on your chair if it isn't high enough for your feet to be off the ground.

Exercise:

Rotate the knee SLOWLY and raise the ankle weight as high as you can.

Hold, then SLOWLY lower down.

**
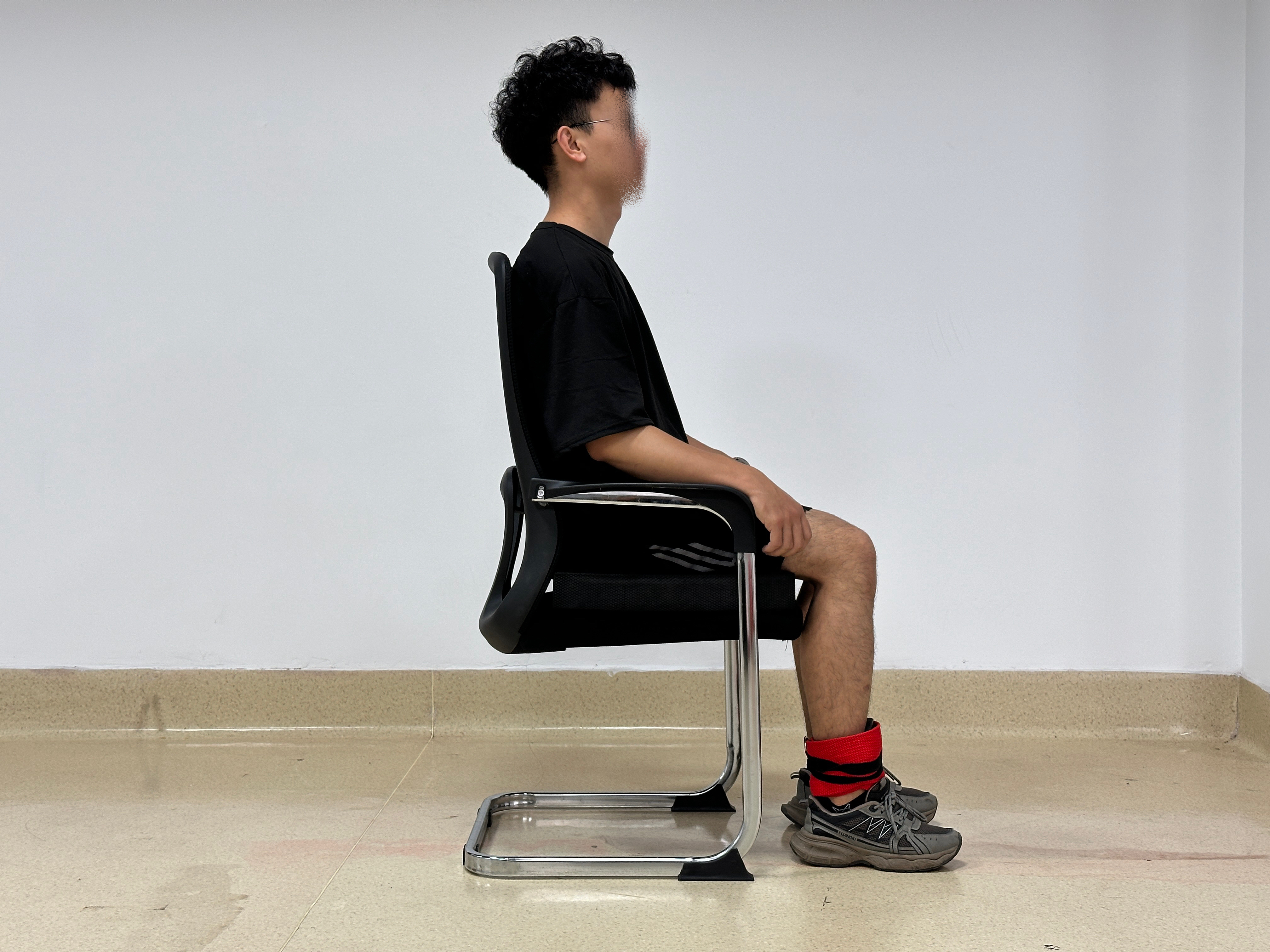

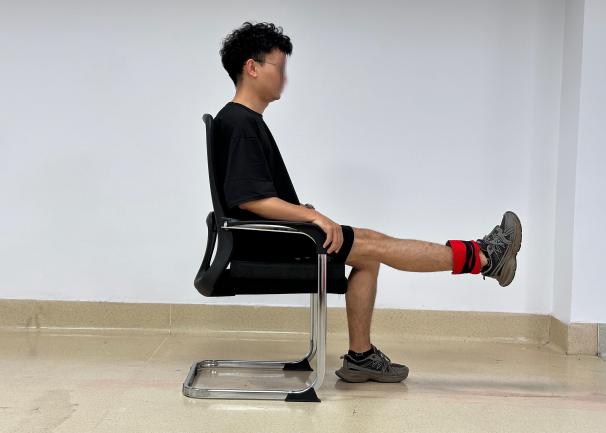
**

**3. Knee extension with hold at 30°**

Starting position:

Put weight on the arthritic leg's ankle.

Sit with your legs hanging off the edge of a bed or chair, and if your chair isn't high enough to elevate your feet, you can place some phone books on the chair.

Exercise:

Straighten your knee and gradually raise your ankle weight up to approximately 30° before fully extending your knee (leaving it slightly bent).

Hold, then SLOWLY lower down.

**
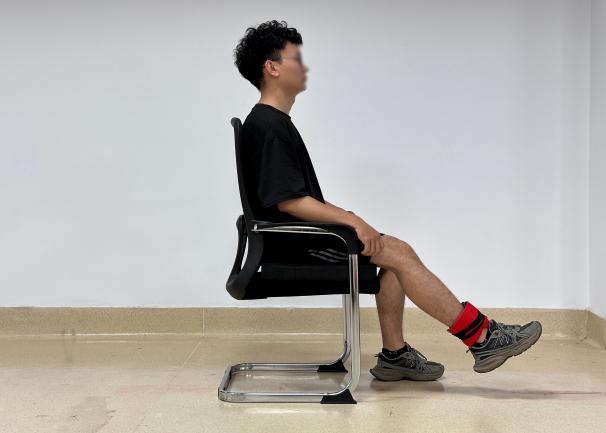

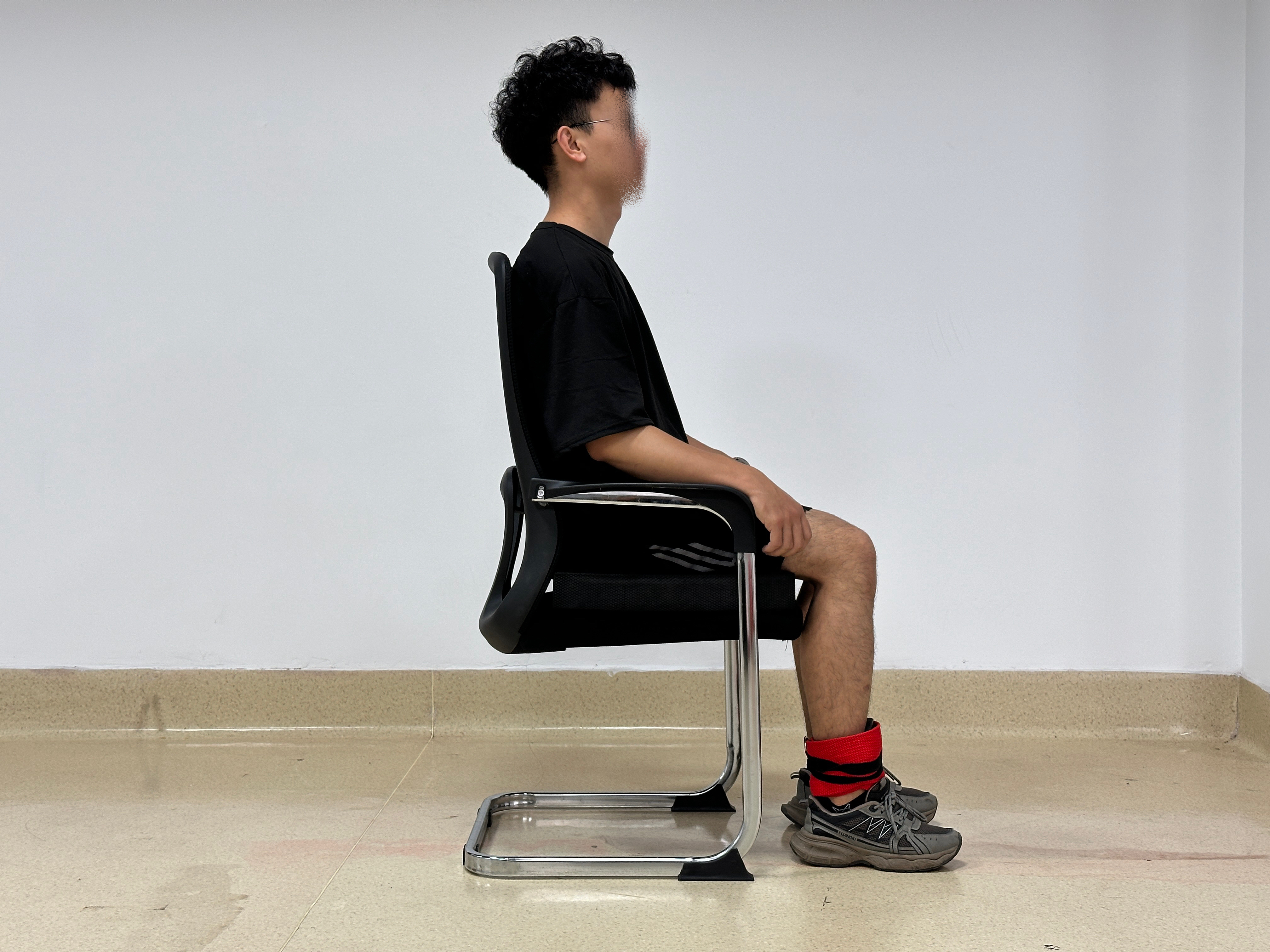
**

**4. Straight Leg Raise**

Starting position:

Put weight on the arthritic leg's ankle.

Lie on your back with your arms and elbows supporting your body weight. Alternatively, feel free to lie down if you prefer.

Bend the non-study leg and maintain the straightness of the arthritic leg, while drawing the ankle and foot towards you.Both the kneecap and toes should be pointing towards the ceiling.

Exercise:

SLOWLY raise your leg until it is about 30 cm or 12 inches off the bed. Make sure to keep your knee straight.

Hold, then SLOWLY lower down.

**
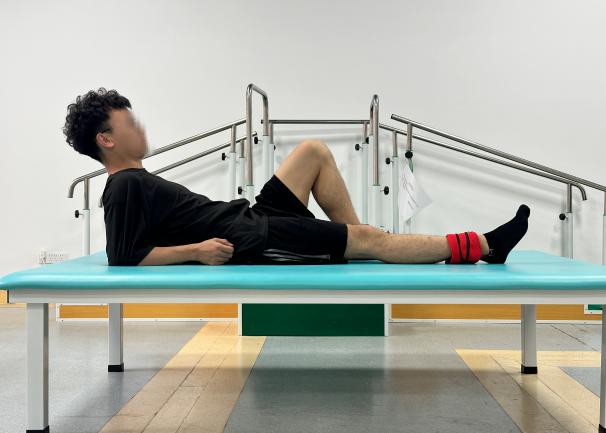

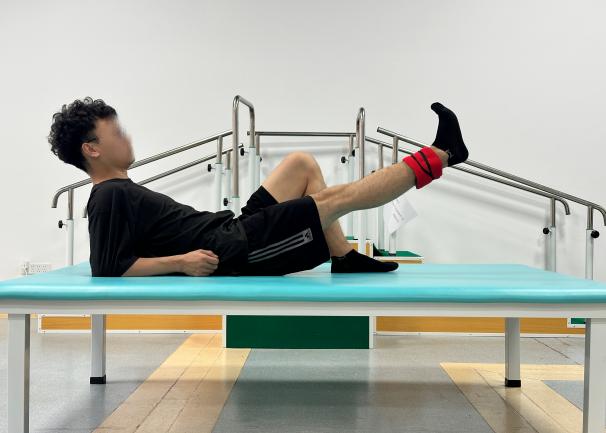
**

**5. Hip Abduction Exercises**

Starting position:

Side lie on your unaffected side, bend the unaffected leg and keep the arthritic leg straight.

Exercise:

SLOWLY raise your leg until it is about 30 cm off the bed. Make sure to keep your knee straight.

Hold, then SLOWLY lower down.

**
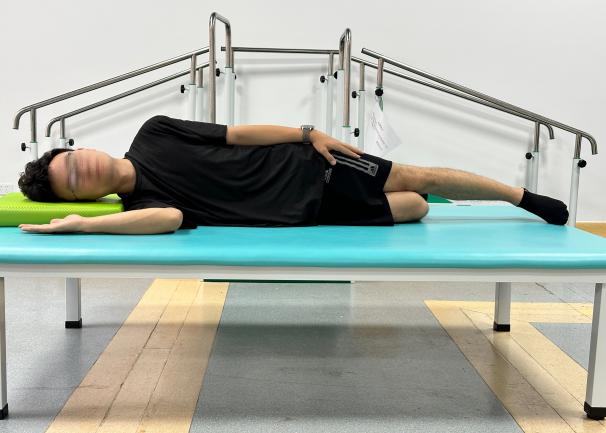

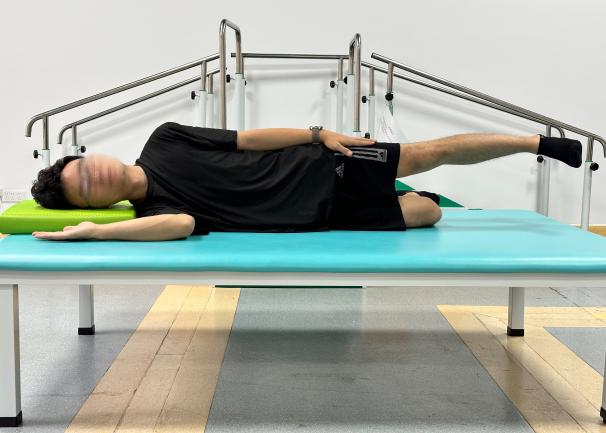
**

**6. Hip Adduction Exercises**

Starting position:

Side lie on your affected side, and keep the arthritic leg straight. The unaffected leg crossover the arthritic leg, and place the foot in front of the arthritic thigh.

Exercise:

SLOWLY raise your leg until it is about 30 cm off the bed. Make sure to keep your knee straight.

Hold, then SLOWLY lower down.

**
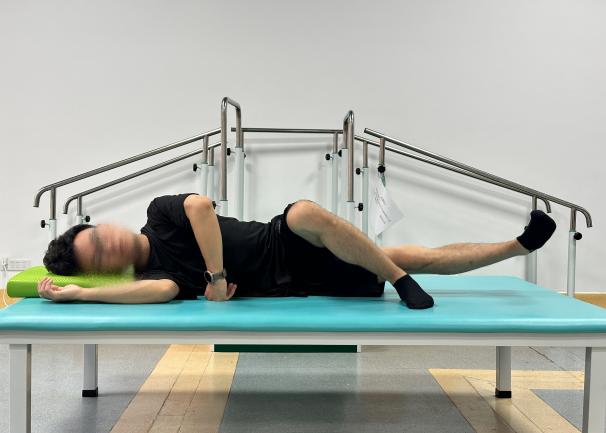

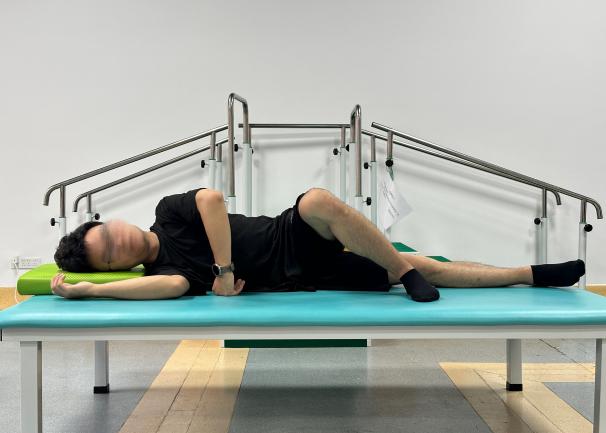
**

**7. Short Arc Knee Extension**

Starting position:

Your physiotherapist will provide you with a rubber band tied into a loop. Place the looped rubber band around the leg of a chair.

Sit on the chair and insert your leg into the looped rubber band.

Exercise:

Slowly straighten your leg towards the rubber band until you experience a comfortable resistance (should be about 60° knee bend).

Hold.

Slowly return to the starting position.

**
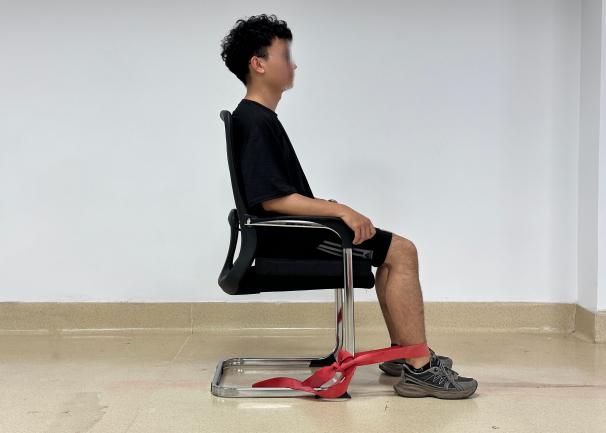

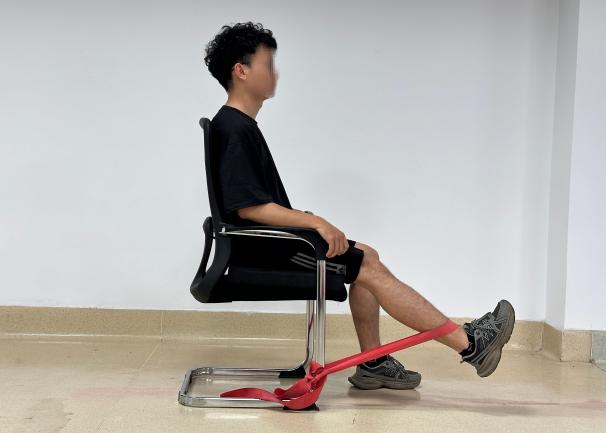
**

**30°**

**30°**

**Supplementary Figure 1: Lower limb strengthening exercise program**
